# Improved rapid and efficient hairy root transformation using *Rhizobium rhizogenes* in legume crops

**DOI:** 10.5511/plantbiotechnology.25.0213a

**Published:** 2025-09-25

**Authors:** Masato Araragi, Pongpan Songwattana, Neung Teaumroong, Sachiko Masuda, Arisa Shibata, Ken Shirasu, Yasuyuki Kawaharada

**Affiliations:** 1 Department of Plant BioSciences, Faculty of Agriculture, Iwate University, Morioka, Iwate 020-8133, Japan; 2 Institute of Research and Development, Suranaree University of Technology, Nakhon Ratchasima 30000, Thailand; 3 School of Biotechnology, Institute of Agricultural Technology, Suranaree University of Technology, Nakhon Ratchasima 3000, Thailand; 4 RIKEN Center for Sustainable Resource Science, RIKEN-TRIP, Yokohama, Kanagawa 230-0045, Japan

**Keywords:** cowpea, mungbean, *Rhizobium rhizogenes*, soybean, transgenic hairy root

## Abstract

Hairy root transformation mediated by *Rhizobium rhizogenes* is a widely used tool for molecular analysis and root material for secondary metabolite production. However, this method is time-intensive, technically demanding, and exhibits low transformation efficiency. To address these limitations, we developed a rapid and efficient hairy root transformation system for legume crops, optimizing protocols with the soybean (*Glycine max* L. Merrill) cultivar Fukuyutaka. Sterilizing seeds with vapor of 5% sodium hypochlorite and germinating them in a double-tier container resulted in over 90% healthy, straight seedlings ideal for transformation, with 3- to 5-day-old seedlings showing the highest transformation rates. Exposing the plant shoot during co-cultivation by covering only the injection area, combined with low nitrogen levels in the hydroponic solution, significantly enhanced hairy root production, yielding up to 16 transgenic hairy roots per plant. Additionally, low nitrogen concentrations were crucial for promoting nodule formation in transgenic hairy roots. These optimized conditions were validated across 12 soybean, 1 cowpea, and 1 mungbean cultivars. The protocol’s effectiveness was confirmed through the induction of symbiotic gene expression of *GmEnod40a* and *GmErn1b* using a promoter β-glucuronidase (GUS) reporter system in transgenic hairy roots. Expression of these genes was detected in both premature and mature nodules, while *GmErn1b* expression was also observed in epidermal cells during early nodulation. This optimized hairy root transformation protocol, requiring under 22 days from seed sterilization to transgenic root induction and 61 days to expression analysis, offers a promising approach for efficient gene function studies in legume crops.

## Introduction

Legume crops, including soybean (*Glycine max* L. Merrill), cowpea (*Vigna unguiculata* L. Walp), and mungbean (*V. radiata* L. Wilczek), are among the most economically significant crops globally, especially in south Asia, and Africa (Nair et al. 2023; Salord et al. 2024). These crops provide essential resources such as human food, animal forage, vegetable oils, and raw materials for various industries (Voisin et al. 2014). Moreover, legumes possess a remarkable ability to convert atmospheric nitrogen into ammonium through symbiosis with soil bacteria, rhizobia, within specialized root nodules. This biological nitrogen fixation plays a vital role in sustainable agriculture by reducing the need for synthetic nitrogen fertilizers, enhancing soil fertility, supporting eco-friendly crop production, and emitting fewer greenhouse gases (GHG) compared to other crops (Stagnari et al. 2017).

Stable plant transformation mediated by *Rhizobium tumefaciens* offers notable advantages in accelerating breeding and plays a vital role in understanding fundamental biological phenomena. Although this technology has been developed and refined since its introduction in 1976 (De Cleene and De Ley 1976), its efficiency remains limited, particularly soybean is only small number of cultivars have been successfully transformed (Freitas-Alves et al. 2024). Moreover, the extended duration required for generating transgenic plants poses a significant limitation, making the process time-consuming and less feasible for high-throughput applications.

Hairy root transformation mediated by *R. rhizogenes*, which targets specific plant tissues, is especially useful for species that are challenging to transform systemically with *A. tumefaciens*. Hairy roots are induced when the *rol* (root loci) genes on the Ri plasmid carried by *R. rhizogenes* are expressed in the host plant cells, and triggering cell division (White et al. 1985). During this process, T-DNA from the Ri plasmid is transferred into the host plant genome via border sequences, enabling the development of transgenic hairy roots (Wang et al. 1984).

This advanced transformation technique is celebrated for its ability to efficiently induce genetically stable, fast-growing hairy roots. Widely applied in root studies, it offers unparalleled utility for analyzing root-specific gene functions and exploring interactions with soil microorganisms, such as bacteria, fungi, and nematodes (Guimaraes et al. 2017; Ruman and Kawaharada 2023). Furthermore, it is highly effective for large-scale production of secondary metabolites in medicinal plants, further cementing its status as a powerful tool in plant biotechnology (Gutierrez-Valdes et al. 2020; Malarz et al. 2023).

Hairy root transformation is adaptable to a broad range of plants and crops. In soybean, two predominant methods are extensively used. The first method, referred to as the *R. rhizogenes*-mediated one-step transformation, involves immersing the cut hypocotyl of a seedling in an *R. rhizogenes* suspension (Fan et al. 2020b). The infected seedling is then incubated in a high-humidity environment for several weeks, facilitating the emergence of hairy roots from the cutting site. The second method requires excising the hypocotyl-end of a 1-day-old seedling, followed by dipping it in an *R. rhizogenes* suspension (Cheng et al. 2021). After around one week of co-cultivation, the cotyledon is transferred to a root growth medium, where hairy roots begin to develop. Both methods typically require approximately 22 to 23 days from seed sterilization to the emergence of transgenic roots, showcasing their efficiency and reliability (Chen et al. 2024; Huang et al. 2022; Li et al. 2017; Niazian et al. 2022; Pereira et al. 2023; Song et al. 2021).

Despite their utility, these methods face challenges, including the frequent occurrence of non-transformed roots alongside transformed ones. Further improvements are necessary to enhance transformation efficiency and reduce the proportion of non-transformed roots, making the process more reliable and scalable for research and biotechnological applications. Hairy root transformation mediated by *R. rhizogenes* has not yet been developed into a complete system applicable to a wide range of soybean cultivars and legume crops. To address this, we conducted a series of optimizations, including seed sterilization and germination, timing of *R. rhizogenes* injection into seedlings, co-culture conditions, and nitrogen content during growth and nodulation, to establish a rapid and efficient hairy root transformation system broadly applicable to legume crops.

## Materials and methods

### Plants and bacteria

Twelve soybean cultivars (*Glycine max* cv. Fukuyutaka, CNS, Enrei, Ezomidori, Hardee, IAC-2, Karasumame, Lee, Ooyukimidori, Peking, Williams 82, Sakkei 4), one cowpea (*Vigna unguiculata* cv. Blackeye), and one mungbean (*V. radiata* cv. KPS1) were used in this study. These legumes crops are used for commercial and research purposes. Before use, seeds were stored under low temperature and dry condition for 1 to 3 years after harvest. A microsymbiont, *Bradyrhizobium diazoefficiens* USDA110 was employed for nodulation analysis. For hairy root transformation, the *Rhizobium rhizogeneis* strain, AR11193, LBA1334, and K599 were tested. *R. rhizogenes* strain K599 were two types: an original K599 strain (NCPPB2659) and a commercially strain with conferred rifampicin resistance. We used the original K599 strain confirmed by genome sequence (Combard et al. 1987; Su et al. 2024; Tong et al. 2018). All bacteria were re-streaked from −80°C freeze stocks on the bacterial medium when we used it.

### Vector constructs

All putative promoter regions and β-glucuronidase (GUS) gene were amplified by PCR using PrimeSTAR® Max DNA Polymerase (Takara Bio Inc. Shiga, Japan), specific primer sets, and the *G. max* cv. Williams 82 genomic DNA isolated using CTAB method from primary leaves (Murray and Thompson 1980) or pIV10::*Ern1* prom.::GUS (Kawaharada et al. 2017) as a template (Supplementary Table S1). The amplified fragments were cloned into a linearized pUB-GFP (35S prom.::GFP) vector (Maekawa et al. 2008), digested with *BamH*I and *Pst*I (New England Biolabs Inc.), using an In-Fusion® HD Cloning Kit (Takara Bio Inc.). The resulting construct vectors in *Escherichia coli* DH5α were transformed to *R. rhizogenes* K599 by triparental mating with *E. coli* MT616 carrying the mobilizing plasmid pRK600 (Finan et al. 1986). *R. rhizogenes* K599 strain carrying pUB-GFP::*GmUbi3* prom.::GUS, pUB-GFP::*GmErn1b* prom.::GUS, and pUB-GFP::*GmEnod40a* prom.::GUS were cultured on Luria–Bertani (LB) medium containing 1.5% (w/v) agar with kanamycin (100 µg/ml) and chloramphenicol (30 µg/ml) at 28°C for 2 days.

### Seed sterilization

Seed sterilization was performed by placing the seeds in one half of a separate Petri dish (φ90×15 mm; AS ONE Co.), adding filter paper soaked with 5% sodium hypochlorite solution (Fujifilm Wako) to the other half. The Petri dish was then sealed, and seeds were exposed to vaper released from the sodium hypochlorite solution for over-night at a room (Supplementary Figure S1A). After sterilization, the seeds were washed three times with sterilized water and sown in a double-tier container (W30.6 × D8.5 × H7.6 cm) (D5294; Sanadaseiko Co. Ltd., Osaka, Japan) containing sterilized vermiculite (Large size; VS-kakou Co. Ltd., Tokyo, Japan) in the upper tier and sterilized water in the lower tier. Water was drawn up through the wick of an alcohol lamp (Maruemu Corporation Co. Ltd., Osaka, Japan) (Supplementary Figure S1B). For sowing, 3.5 cm of vermiculite was laid down, after which the seeds were placed and covered with 2 cm of dry vermiculite. In the single-tier container, sterilized vermiculite was placed, and thoroughly soaked with sterilized water before sowing seeds. Seeds were incubated in the growth chamber (LPH-411PFQDT-S; Nippon Medical & Chemical Instruments Co., Ltd., Tokyo, Japan) at 28°C (16 h light, 20% LED irradiation/8 h dark) for 2 to 8 days.

### Injection of *R. rhizogenes* and co-cultivation

*R. rhizogenes* K599 grown on an LB agar plate was injected into the upper hypocotyl of early seedlings at two to six spots using a 0.4 mm diameter needle tip (27G; Terumo Co.) (Supplementary Movie S1). For co-cultivation, the injected seedling was transplanted into a sterilized double-magenta box (Bio Medical Science Inc.) containing sterilized vermiculite (No. 2; VS-kakou Co., Ltd.) and soybean hydroponic solution (Araragi et al. 2021) with appropriate nitrogen concentrations (KNO_3_ and NH_4_Cl). The setup was covered with a sterilized, ventilated magenta box (Bio Medical Science Inc.) and incubated for 4 days at 28°C (16 h light/8 h dark) (Supplementary Figure S1D). Subsequently, the ventilated magenta box was removed and only the injection area of *R. rhizogenes* was covered with aluminum foil, exposing the plant shoot (Supplementary Figure S1E–G). The seedlings were then maintained for 14 days at 28°C (16 h light/8 h dark), allowing hairy roots to be induced from the injection area covered with aluminum foil (Supplementary Figure S1F, G).

### Inoculation of bradyrhizobia

The *B. diazoefficiens* USDA110 was re-streaked onto the arabinose-gluconate (AG) medium (Sadowsky et al. 1987) containing 1.5% agar and incubated for 7 days at 28°C. For preparing the bradyrhizobial inoculant, a single colony of *B. diazoefficiens* USDA110 was cultured in a conical flask with the liquid AG medium for 5 days at 28°C.

Seedlings with grown hairy roots were transplanted into a new double-magenta box containing soybean hydroponic solution with low nitrogen concentrations (1 mM KNO_3_ and 0.1 mM NH_4_Cl). Seven days later, *B. diazoefficiens* USDA110, diluted with 50 ml of sterilized water per magenta box to OD_600_=0.04, was inoculated. The plants were then incubated for 14 to 21 days at 25°C during the day and 21°C at night (16 h light/8 h dark).

### GUS staining

Fresh plant tissues were soaked in GUS staining buffer (0.5 mg/ml 5-bromo-4-choloro-3-indolyl-β-d-glucuronide (X-Gluc), 100 mM NaPO_4_ pH7.0, 10 mM EDTA pH8.0, 1 mM K_3_[Fe(CN)_6_], 1 mM K_4_[Fe(CN)_6_]·3H_2_O, 0.1% Triton-X-100) for 10 to 34 h at 37°C. After staining, root and nodule samples were observed under a Leica M165 FC microscope.

## Results

### Improved hairy root transformation

**Figure 1:**
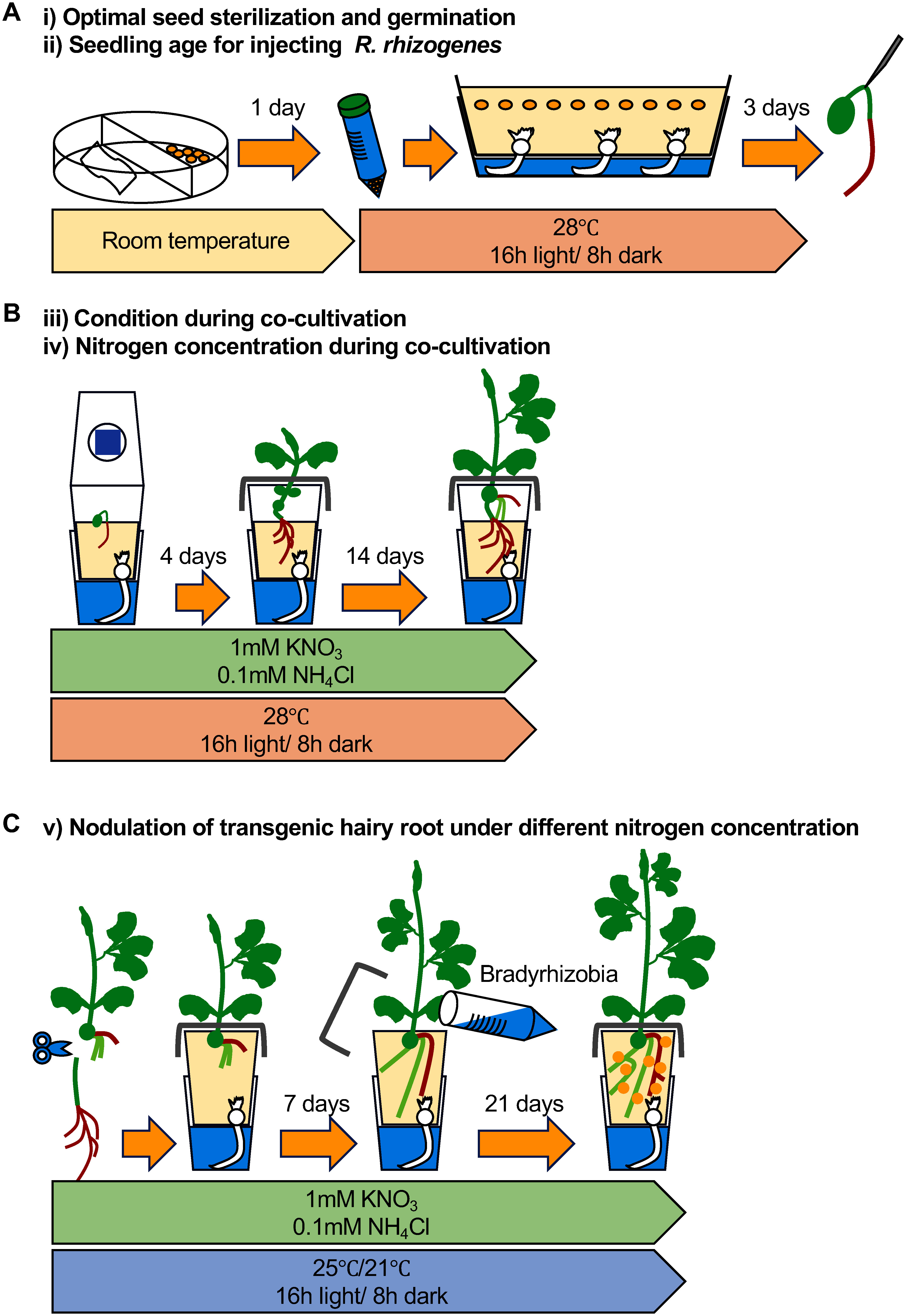
Figure 1. Outline of rapid and efficient hairy root transformation method. A, schematic representation of seed sterilization, germination, and *Rhizobium rhizogenes* injection. B, schematic representation of cocultivation. C, schematic representation of nodule formation under varying nitrogen concentrations.

To establish a rapid and high-efficiency hairy root transformation system in legume crops, we performed a series of optimizations using *G. max* cv. Fukuyutaka. These included seed sterilization and germination, timing of *R. rhizogenes* injection, co-cultivation conditions, and nitrogen content throughout the co-cultivation and Bradyrhizobia inoculation process (detailed below from i) to v)). Consequently, we developed an improved hairy root transformation protocol, as illustrated in Figure 1.

#### i) Optimal seed sterilization and germination

**Figure 2:**
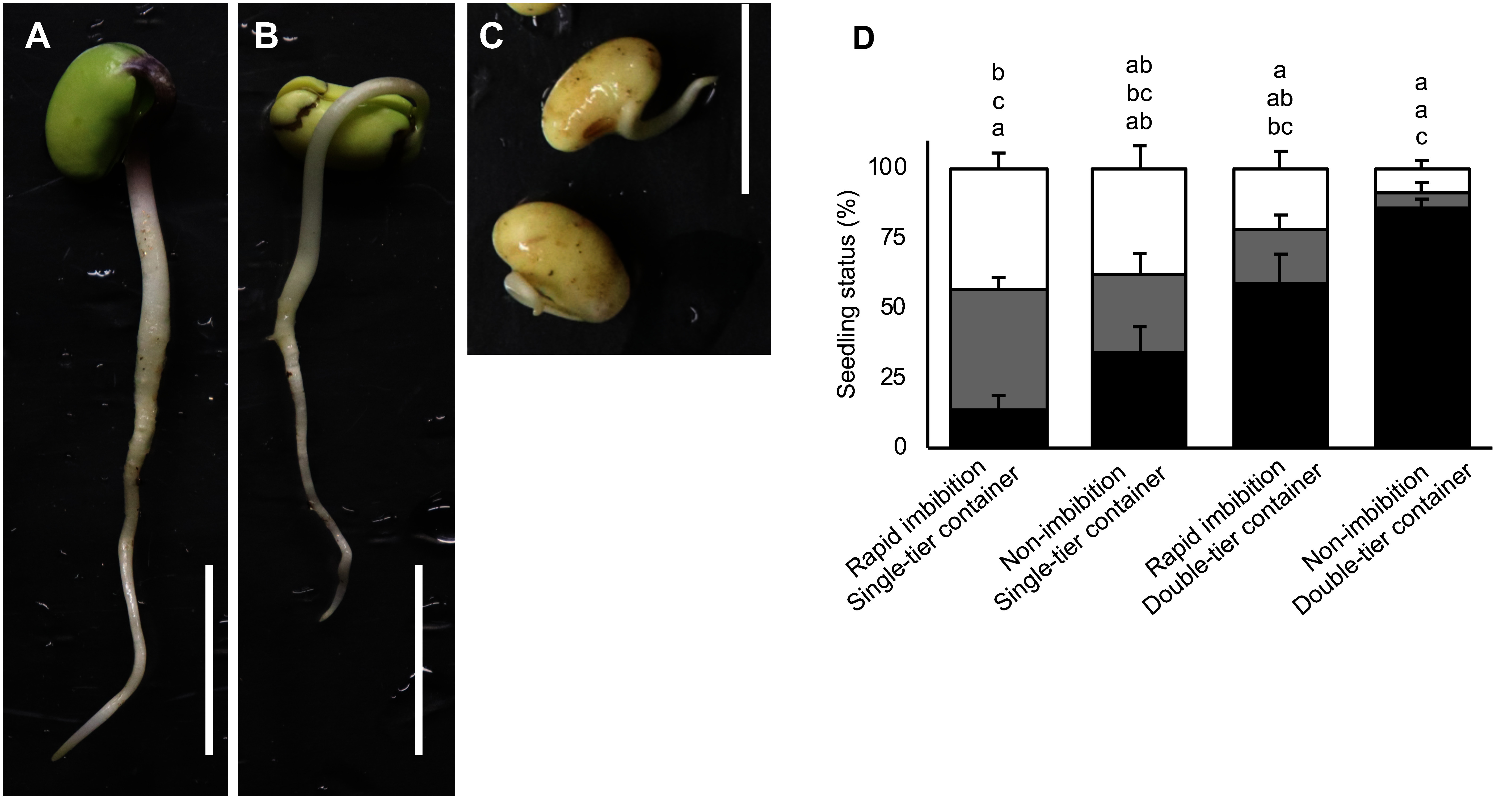
Figure 2. Germination status of soybean seedlings. The germination status of 3-day-old *Glycine max* cv. Fukuyutaka seedlings was categorized as follows: A, healthy and straight seedlings; B, slightly damaged seedlings; and C, abnormally germinated seedlings or non-germinated seeds. Scale bars represent 2.0 cm. D, the ratio of 3-day-old seedling status under different germination conditions. Black bars represent healthy seedlings, as in A; gray bars represent slightly damaged seedlings, as in B; and white bars represent abnormally germinated seedlings or non-germinated seeds, as in C. Means and standard errors are derived from three independent experiments (*n*=20 in each condition). Letters above each bar indicate statistically significant differences by Tukey–Kramer’s HSD test at *p*<0.05.

For the injection of *R. rhizogenes*, healthy, non-damaged, and straight-growing soybean seedlings are required (Figure 2A). Since rapid seed imbibition can lead to incomplete germination (Sato et al. 2019; Sayama et al. 2009), we examined the effect of seed imbibition before sowing. Seeds imbibed for 10 min resulted in 59.2±10.2% healthy and straight seedlings, 19.2±5.1% slightly damaged seedlings, and 21.7±6.1% abnormal seedlings or non-germination (Figure 2A–C). In contrast, seeds sown without imbibition germinated 85.9±3.3% healthy and straight seedlings in a double-tier container (Figure 2, Supplementary Figure S1B). Additionally, using a single-tier container for germination resulted in fewer than 40% healthy and straight seedlings due to rapid imbibition (Figure 2D). These findings indicate that rapid imbibition significantly adversely affects soybean seed germination.

To prevent rapid imbibition, seed sterilization conditions were evaluated. Seeds were sterilized overnight with vapor of 5% sodium hypochlorite in a separate Petri dish, achieving a germicidal effect comparable to that of 1% (v/v) sodium hypochlorite solution (Supplementary Figures S1A, S2). The germination status of seeds treated with vapor of 5% sodium hypochlorite was superior to those treated with 1% sodium hypochlorite solution or non-sterilized seeds (Supplementary Figure S3). To confirm the effectiveness of vapor of 5% sodium hypochlorite sterilization for sowing without imbibition, an experiment was conducted using 11 soybean and 2 Vigna cultivars. Except for Ezomidori, all cultivars produced healthy and straight seedlings, with slightly damaged seedlings exceeding 80%, making them suitable for *R. rhizogenes* injection (Table 1, Supplementary Figure S4).

**Table 1:** Table 1. Available seedling ratio in soybean and Vigna cultivars.

Cultivar	Tested seeds	Available seedlings*^1^	Ratio (%)
*G. max* cv. Fukuyukata	710	661	93.1
*G. max* cv. CNS	40	38	95.0
*G. max* cv. Enrei	70	69	98.6
*G. max* cv. Ezomidori	45	31	68.9
*G. max* cv. Hardee	153	142	92.8
*G. max* cv. IAC-2	60	49	81.7
*G. max* cv. Karasumame	120	106	88.3
*G. max* cv. Lee	45	43	95.6
*G. max* cv. Ooyukimidori	45	42	93.3
*G. max* cv. Peking*^2^	40	32	80.0
*G. max* cv. Williams 82	80	79	98.8
*G. max* cv. Sakkei 4	50	45	90.0
*V. unguiculata* cv. black eye	70	56	80.0
*V. radiata* cv. KPS1	60	55	91.7

Experiments were repeated at least three times. *^1^ Available seedling indicates healthy seedlings and slightly damaged seedlings as described in Figure 2A, B. *^2^ Seed coat was scratched before sterilization.

#### ii) Seedling age for *R. rhizogenes* injection

**Figure 3:**
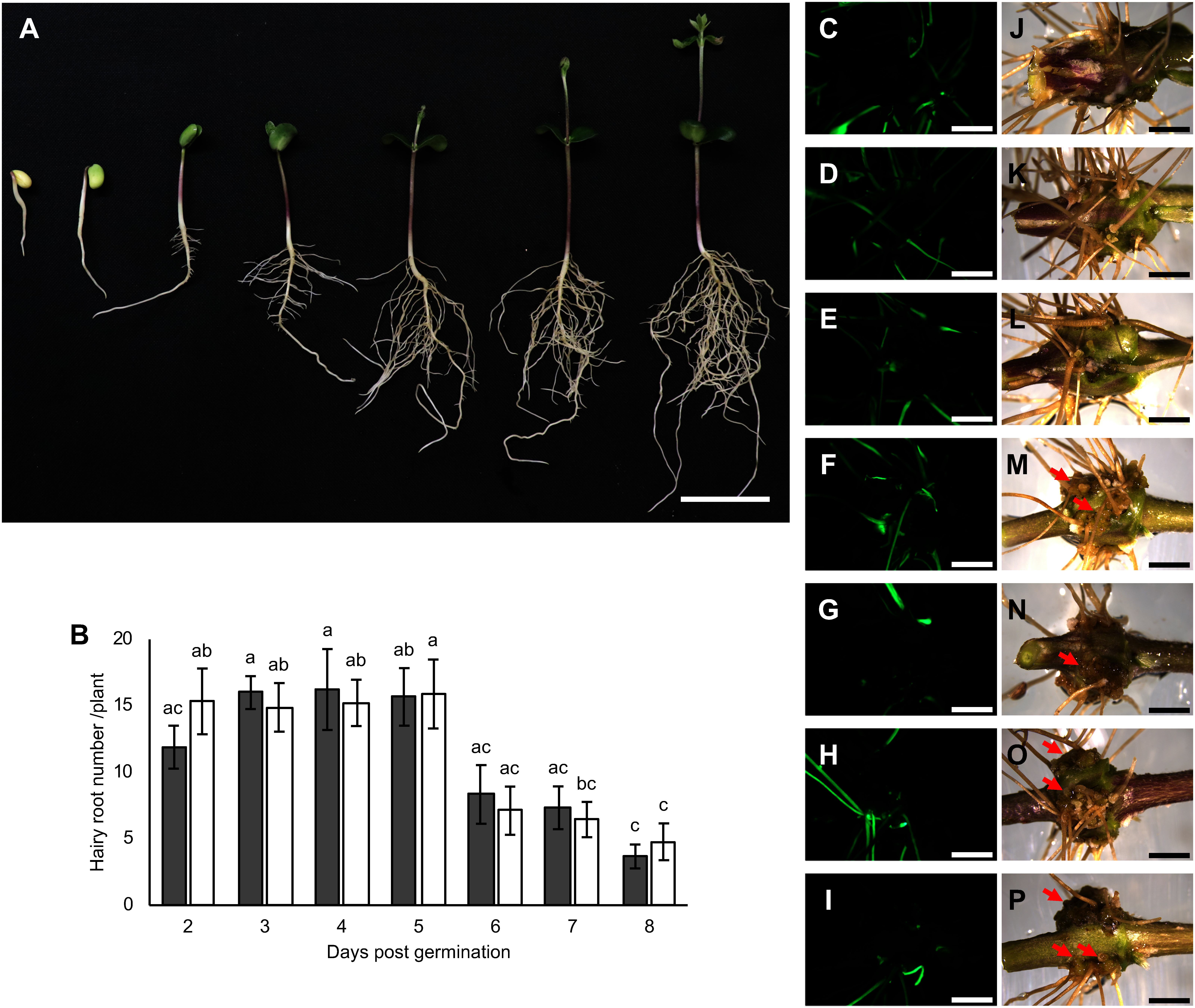
Figure 3. Hairy root induction in seedling of different ages. A, status of 2- to 8-day-old seedlings used for *Rhizobium rhizogenes* injection. Scale bars represent 5.0 cm. B, the average number of transgenic and non-transgenic hairy roots including adventitious roots longer than 1 cm. Root numbers were counted across all tested plants, as shown in Supplementary Table S2. Gray bars represent green fluorescent protein (GFP)-expressing transgenic hairy roots, and white bars represent non-GFP-expressing non-transgenic hairy roots including adventitious roots. Means and standard errors are based on three independent experiments (*n*=9). Letters above each bar indicate statistically significant differences by Tukey–Kramer’s HSD test at *p*<0.05. C to P, hairy root induction was observed 18 days after injection for seedlings of various ages: 2-day-old seedlings in C and J, 3-day-old seedlings in D and K, 4-day-old seedlings in E and L, 5-day-old seedlings in F and M, 6-day-old seedlings in G and N, 7-day-old seedlings in H and O, and 8-day-old seedlings in I and P. C to I show GFP fluorescence, and J to P show bright-field images. Red arrows indicate callus-like tissue formations. Scale bars indicate 0.5 cm.

To evaluate the usability of *R. rhizogenes* strains, hairy root transformation was performed using the AR11193, LBA1334, and K599. Unlike the K599 strain, AR11193 and LBA1334 did not produce transgenic hairy root. To determine the optimal timing for *R. rhizogenes* injection into the upper hypocotyl, we conducted experiments using seedlings of various ages (Figure 3A) with *R. rhizogenes* K599 carrying pUB-GFP::*GmUbi3* prom.::GUS. After injection, the plants were cultivated according to the conditions outlined in Figure 1A. Transgenic hairy roots were produced after approximately 12 days post-injection (Supplementary Figure S5A–C), and identified GFP fluorescence under a fluorescence microscope and by GUS staining with X-Gluc at 18 days post-injection. A transgenic hairy root showed heterogeneously expressions of GUS and GFP (Supplementary Figure S5D–F). Due to differences in insertion loci and the activity of *GmUbi3* or *35S* promoters, GFP and GUS expression in transgenic hairy roots were not completely much each other (Supplementary Figure S5G). Approximately 50% of total hairy roots per plant exhibited GFP expression roots (Figure 3B, Supplementary Figure S5G). Our findings show that 3- to 5-day-old seedlings produced 15.7±2.2 to 16.2±3.0 transgenic roots per plant, significantly higher than the 8.3±2.2 to 3.7±0.9 roots per plant observed with 6- to 8-day-old seedlings, respectively (Figure 3B, Supplementary Table S2). Additionally, callus-like tissue formation was observed at the injection sites of 5- to 8-day-old seedlings (Figure 3M–P).

#### iii) Condition during co-cultivation

**Figure 4:**
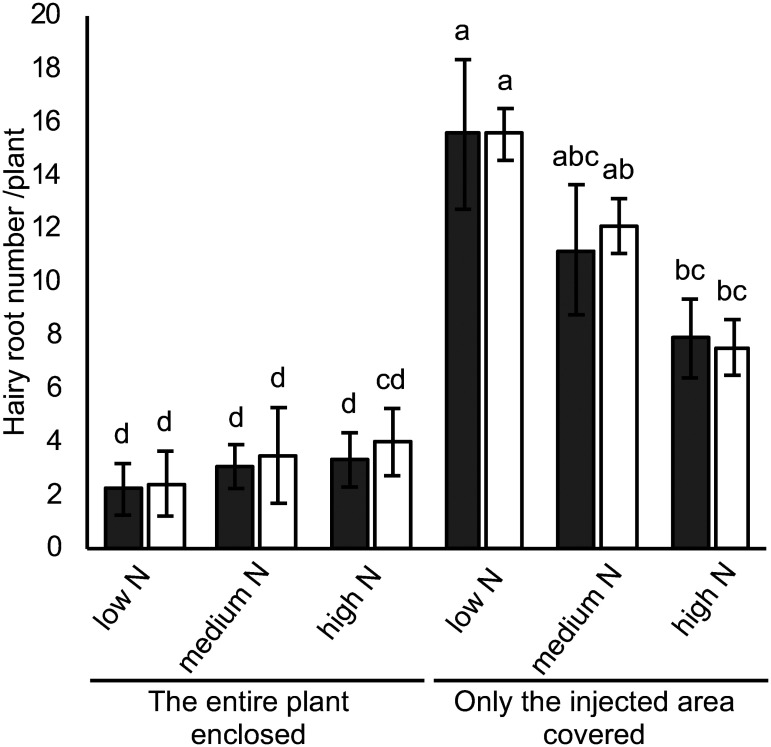
Figure 4. Hairy root inductions during different co-cultivation condition. Three-day-old *Glycine max* cv. Fukuyutaka seedlings injected with *Rhizobium rhizogenes* were incubated either with the entire plant enclosed or with only the injected area covered under low (1 mM KNO_3_ and 0.1 mM NH_4_Cl), medium (5 mM KNO_3_ and 0.5 mM NH_4_Cl), and high (10 mM KNO_3_ and 1 mM NH_4_Cl) nitrogen conditions. Each bar represents the average number of transgenic and non-transgenic hairy roots including adventitious roots longer than 1 cm, 18 days after injection. The gray bar represents green fluorescent protein (GFP)-expressing transgenic hairy roots, and the white bar represents non-GFP-expressing non-transgenic hairy roots including adventitious roots. Means and standard errors are derived from three to four independent experiments (*n*=9 to 12). Letters above each bar indicate statistically significant differences by Tukey–Kramer’s HSD test at *p*<0.05.

In previous hairy root inductions, whole plants and tissues were enclosed after co-cultivation with *R. rhizogenes* to maintain the high humidity (Cheng et al. 2021; Fan et al. 2020a, b; Ji et al. 2019; Kereszt et al. 2007). Based on these methods, we initially covered 3-day-old seedlings injected with *R. rhizogenes* K599 using a magenta box (Supplementary Figure S1C). However, the number of GFP-expressing hairy roots was low, averaging from 2.2±1.0 to 3.3±1.0 per plant (Figure 4) and some plants failed to produce any hairy roots (Supplementary Table S3). To improve hairy root induction efficiency, we modified the setup by covering the injected seedlings with a ventilated magenta box for 4 days, followed by covering only the injection area with aluminum foil, leaving the plant shoot exposed (Supplementary Figure S1D, E). This modification resulted in a more than 3.4-fold increase in the number of hairy roots compared with the closed condition (Figure 4).

#### iv) Nitrogen concentration during co-cultivation

Root primordium development and root elongation are known to be influenced by nitrogen concentration in the nutrient medium (Motte et al. 2019; O’Brien et al. 2016; Pélissier et al. 2021). To examine this effect, injected plants were incubated under different nitrogen concentrations: low (1 mM KNO_3_ and 0.1 mM NH_4_Cl), medium (5 mM KNO_3_ and 0.5 mM NH_4_Cl) and high (10 mM KNO_3_ and 1 mM NH_4_Cl). While all plants with the injection area covered by aluminum foil produced transgenic hairy roots with an approximate transformation rate of 50% (Figure 4, Supplementary Table S3), the number of GFP-expressing transgenic hairy roots was significantly higher under low nitrogen conditions compared to medium or high nitrogen conditions (Figure 4). Moreover, this open system combined with low nitrogen conditions effectively induced GFP-expressing transgenic hairy roots in all soybean and Vigna cultivars (Table 2, Supplementary Figure S6). Transformation efficiency was enhanced, resulting in the induction of numerous GFP-expressing transgenic hairy roots.

**Table 2:** Table 2. Transformation ratio in soybean and Vigna cultivars.

Cultivar	Tested plant	Transformed plant*	Ratio
*G. max* cv. Fukuyukata	30	30	100.0%
*G. max* cv. CNS	5	5	100.0%
*G. max* cv. Enrei	5	5	100.0%
*G. max* cv. Ezomidori	5	5	100.0%
*G. max* cv. Hardee	5	5	100.0%
*G. max* cv. IAC-2	5	5	100.0%
*G. max* cv. Karasumame	5	5	100.0%
*G. max* cv. Lee	5	4	80.0%
*G. max* cv. Ooyukimidori	5	5	100.0%
*G. max* cv. Peking	7	7	100.0%
*G. max* cv. Williams 82	5	5	100.0%
*G. max* cv. Sakkei 4	5	5	100.0%
*V. unguiculata* cv. black eye	10	10	100.0%
*V. radiata* cv. KPS1	15	12	80.0%

Experiments was performed at least one time. *Transgenic plant means that has produced one or more GFP-expression transgenic roots.

#### v) Nodulation of transgenic hairy root under different nitrogen concentration

**Figure 5:**
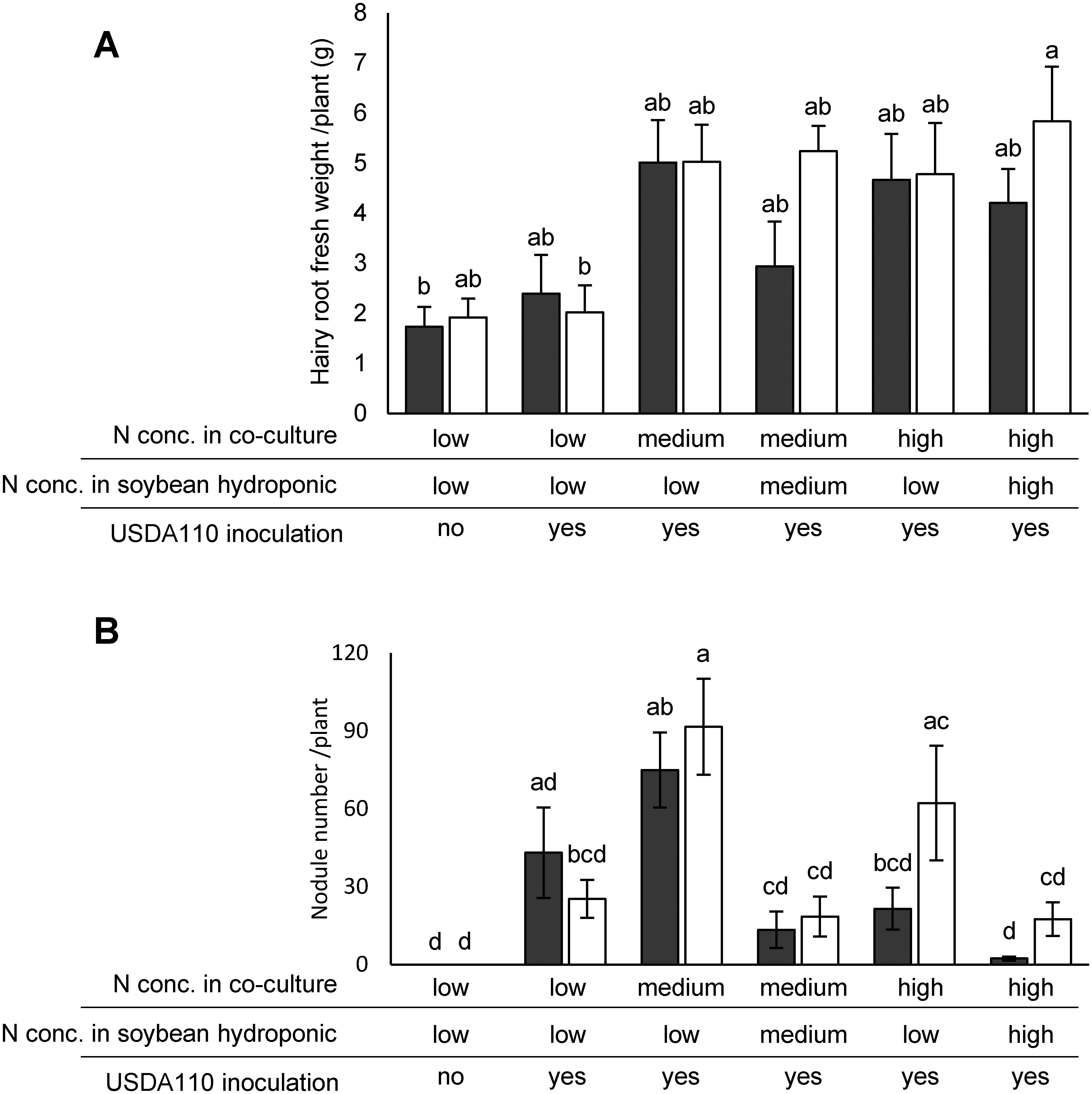
Figure 5. Nodule formation in hairy roots under different nitrogen conditions. Three-day-old *Glycine max* cv. Fukuyutaka seedlings injected with *Rhizobium rhizogenes*, with only the injected area covered, were grown under the same nitrogen conditions described in Figure 4. Seedlings with hairy roots were subsequently transferred to soybean hydroponic solutions containing low (1 mM KNO_3_ and 0.1 mM NH_4_Cl), medium (5 mM KNO_3_ and 0.5 mM NH_4_Cl), or high (10 mM KNO_3_ and 1 mM NH_4_Cl) nitrogen conditions. Each bar represents the average fresh weight of the hairy roots in A and the nodule number in B at 21 days after inoculation with *Bradyrhizobium japonicum* USDA110. These measurements were taken using all tested plants shown in Supplementary Table S4. The gray bar represents green fluorescent protein (GFP)-expressing transgenic hairy roots, and the white bar represents non-GFP-expressing non-transgenic hairy roots including adventitious roots. Means and standard errors are based on one to three independent experiments (*n*=7 to 9). Letters above each bar indicate statistically significant differences by Tukey–Kramer’s HSD test at *p*<0.05.

A high nitrogen concentration in hydroponic solution inhibits nodule symbiosis (Nishida et al. 2018). To evaluate how nitrogen concentration affects nodule formation in transgenic hairy roots, transgenic hairy roots were induced under varying nitrogen concentrations, as shown in Figure 4. The roots were then cut from the hypocotyl, and seedlings with hairy roots were transferred to a new magenta box containing soybean hydroponic solution with different nitrogen concentrations. Seven days after transfer, *B. diazoefficiens* USDA110 was inoculated onto the roots (Figure 5). Although no significant differences were observed in GFP-expressing hairy roots across all bradyrhizobial-inoculated conditions compared to non-GFP-expressing hairy roots including adventitious roots (Figure 5A), the number of nodules was significantly reduced and suppressed in some plants grown under medium and/or high nitrogen concentrations during co-cultivation (Figure 5B, Supplementary Table S4). These results suggest that optimal conditions for nodule symbiosis experiments involve growing plants under low or medium nitrogen concentrations following *R. rhizogenes* injection, with a subsequent transfer to low nitrogen conditions (Figure 5).

### Gene expression of transgenic hairy roots in soybean

**Figure 6:**
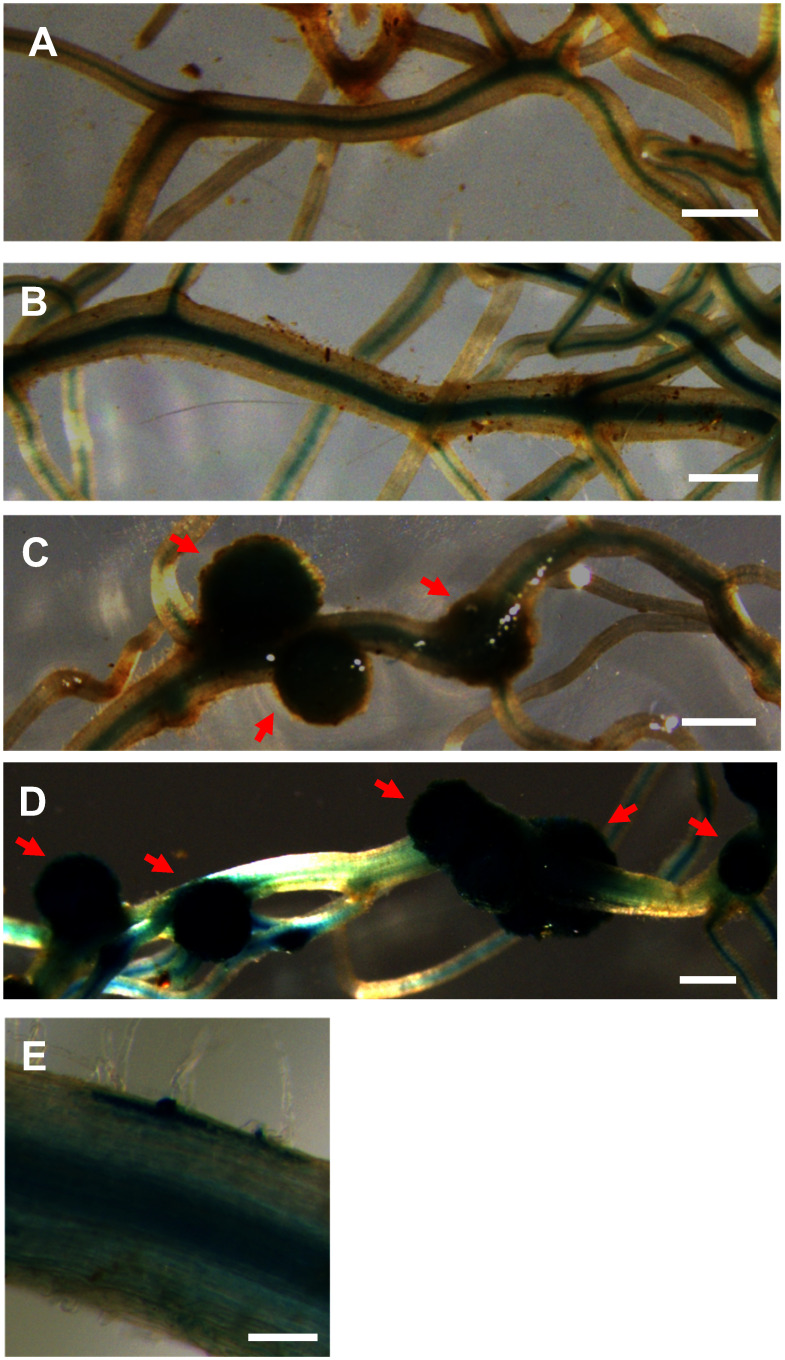
Figure 6. Promoter β-glucuronidase (GUS) assay after *Bradyrhizobium* inoculation. Transgenic hairy roots of *Gycine max* cv. Fukuyutaka were stained using 5-bromo-4-chloro-3-indolyl β-d-glucuronide (X-gluc) 14 days after inoculation with *Bradyrhizobium diazoefficiens* USDA110. *GmEnod40a* prom.::GUS transgenic hairy roots in A and C and *GmErn1b* prom.::GUS transgenic hairy roots in B, D, and E: non-inoculated in A and B, inoculated in C, D, and E. Scale bar indicates 1 mm in A to D and 100 µm in E. Red arrows indicate premature and mature nodules.

To validate the established hairy root transformation method as a research tool, we examined the expression patterns of symbiotic genes, specifically *GmEnod40a* and *GmErn1b* (Song et al. 2022). Constructs pUB-GFP::*GmErn1b* prom.::GUS and pUB-GFP::*GmEnod40a* prom.:GUS were introduced into *G. max* cv. Fukuyutaka using the established hairy root transformation system (Figure 1). Two weeks after inoculation with *B. diazoefficiens* USDA110, GUS activity was analyzed via X-Gluc staining (Figure 6). As a result, *GmEnod40a* and *GmErn1b* expressions were observed in both premature and mature nodules (Figure 6C, D). Additionally, *GmErn1b* expression was detected in an epidermal cell during early nodulation (Figure 6E).

## Discussion

In this study, we established a rapid and efficient *R. rhizogenes*-mediated hairy root transformation system for soybean. Various hairy root transformation methods in soybean were modified and reported. One-step and hypocotyl-end transformations produced 6.5 and 4.5 transgenic hairy roots per plant 22 to 23 days after seed germination (Cheng et al. 2021; Fan et al. 2020b). In this study, the optimized protocol significantly enhanced the transformation rate while reducing the time required to generate transgenic hairy roots compared to conventional methods. Transgenic hairy roots were started to induce 16 days after seed sterilization (Supplementary Figure S5), and approximately 16 transgenic roots per plant within less than 22 days (Figure 3B) underscores its efficiency and practicality for high-throughput applications (Figure 1). Moreover, its successful application to other legume crops, including cowpea and mungbean, demonstrates its versatility and potential for broader use in future research endeavors.

Soybean seed germination is often performed on plates in laboratory settings, with limited emphasis placed on germination methods. However, soybean seeds are highly sensitive to waterlogging, and rapid water absorption can negatively affect seedling quality, causing physical damage and abnormal growth due to nitric oxide accumulation (Araragi et al. 2021; Sanz et al. 2015). In our system, we mitigated the effects of rapid water absorption by using a double-tier container combined with vapor of 5% sodium hypochlorite seed sterilization. This approach achieved over 90% healthy, straight-growing seedlings (Figures 1A, 2). This germination system not only improves overall transformation efficiency but also serves as a broadly applicable method for studies involving seedlings.

The age of seedlings plays a crucial role in achieving high transformation rates (Bandaranayake and Yoder 2018; Wang et al. 2024; Zhang et al. 2024). Our findings revealed that 3- to 5-day-old seedlings generated the highest number of transgenic hairy roots compared to both younger seedlings (2-day-old) and older seedlings (6- to 8-day-old) (Figure 3). Notably, 6- to 8-day-old seedlings exhibited increased callus-like formation at the injection site, potentially interfering with hairy root induction (Figure 3M–P). These observations indicate that 3- to 5-day-old seedlings offer optimal conditions for hormone balance, particularly auxin and cytokinin, and cellular responsiveness required for effective hairy root transformation (Pan et al. 2019; Sun and Zhu 2021).

Maintaining adequate humidity at the injection site during cocultivation is crucial for effective *R. rhizogenes* infection and subsequent hairy root induction (Cheng et al. 2021; Fan et al. 2020a, b; Ji et al. 2019; Kereszt et al. 2007). Many hairy root transformation systems address this by enclosing the entire plant to sustain high humidity; however, soybean seedlings are sensitive to humidity and waterlogging (Kokubun 2013). Enclosing the entire plant often led to chlorosis (data not shown) and reduced hairy root induction efficiency (Figure 4). To overcome these challenges, we modified the system by using ventilated magenta boxes and aluminum foil to confine humidity to the injection site (Figure 1B). This approach not only improved hairy root induction but also promoted healthy shoot growth by avoiding stress from excessive humidity (Figure 4). By balancing the specific requirements of the infection site with overall plant health, the system became both more effective and plant-friendly (Lee et al. 2019).

Nitrogen levels are critical for both root development and nodule formation (Motte et al. 2019; Nishida and Suzaki 2018). In this study, low nitrogen concentrations (1 mM KNO_3_ and 0.1 mM NH_4_Cl) during the co-cultivation period significantly increased the number of hairy roots compared to high nitrogen conditions (10 mM KNO_3_ and 1 mM NH_4_Cl) (Figure 4). Furthermore, high nitrogen levels during co-cultivation adversely affected subsequent nodulation capacity, reducing the ability of nodule formation even when using soybean hydroponic solutions with low nitrogen levels (Figure 5, Supplementary Table S4). These results emphasize the need for a two-phase nitrogen management strategy: applying medium nitrogen concentrations (5 mM KNO_3_ and 0.5 mM NH_4_Cl) during co-cultivation to induce transgenic hairy roots and transitioning to low nitrogen levels for nodulation. This method effectively balances robust hairy root development with successful nodule formation (Figure 5). The strategy offers a reliable framework for studying symbiosis-related genes in legumes (Figure 6) and holds potential for broader application in improving related research systems.

The aspects investigated in this study are not confined to legume crops but are equally important for developing new hairy root transformation systems in a wide range of crop cultivars and plant species.

## Description of supplementary files

Supplementary Table S1. The primer list.

Supplementary Table S2. Number of plant having hairy root.

Supplementary Table S3. Number of plant having hairy root.

Supplementary Table S4. Number of plant having nodules.

Supplementary Figure S1. Equipments for the hairy root transformation.

Supplementary Figure S2. Efficiency of seeds sterilization.

Supplementary Figure S3. Condition of 3-day-seedlings.

Supplementary Figure S4. Three-3-day-seedlings-seedlings of soybean and Vigna cultivars.

Supplementary Figure S5. Green fluorescent protein (GFP)- and β-glucuronidase (GUS)-based transgenic hairy roots.

Supplementary Figure S6. Transgenic hairy roots of soybean and Vigna cultivars.

Supplementary Movie S1. How to inject *R. rhizogenes* to hypocotyl of seedlings.
