# Supplementary material for: Improved rapid and efficient hairy root transformation using Rhizobium rhizogenes in legume crops
Source: Plant Biotechnol (Tokyo). 2025 Sep 25;42(3):279–88. doi: 10.5511/plantbiotechnology.25.0213a (PMC12573540; doi:10.5511/plantbiotechnology.25.0213a)
Supplement: Supplementary Data [file plantbiotechnology-42-3-25.0213a_s001.pdf]

Supplementary Table S1. The primer list

primer name		primer sequence (5' to 3')	PCR product length (bp)
Gus gene for In-Fusion	forward primer	ATGTTACGTCCTGTAGAAAC	2020
	reverse primer	CCTAGGTACCGGATCTCAATCATTGTTTGCCTCCC	
GmEnod40a promoter for In-Fusion	forward primer	AGTGCCAAGCTGGGCGACTGCTACTTTTTT	1343
	reverse primer	TACAGGACGTAACATGCTTCTTCAAGAACCATGG	
GmErn1b promoter for In-Fusion	forward primer	AGTGCCAAGCTGGGCCAACAAATATAAAAA	5804
	reverse primer	TACAGGACGTAACATCACAATTTTATTGAAC	
GmUbi3 promoter for In-Fusion	forward primer	AGTGCCAAGCTGGGCCTTTTAGTCTTATGTTATTG	1475
	reverse primer	TACAGGACGTAACATCTGTCGAGTCAACAATCAC	

Supplementary Table S2. Number of plant having hairy root

	tested plants	plant with hairy root
2-day-old seedling	9	9
3-day-old seedling	9	9
4-day-old seedling	9	9
5-day-old seedling	9	9
6-day-old seedling	9	8
7-day-old seedling	9	8
8-day-old seedling	9	9

Supplementary Table S3. Number of plant having hairy root

condition	N concentration	tested plants	plants with hairy root
The entire plant enclosed	low N	9	7
	medium N	12	9
	high N	9	7
Only the injected area covered	low N	11	11
	medium N	9	9
	high N	9	9

Supplementary Table S4 number of plant having nodules

N conc. in soybean hydroponic	N conc. in soybean hydroponic	USDA110 inoculation	tested plant	plant induced nodule
low	low	No	7	0
low	low	yes	8	8
medium	low	yes	9	9
medium	medium	yes	9	7
high	low	yes	9	7
high	high	yes	9	7

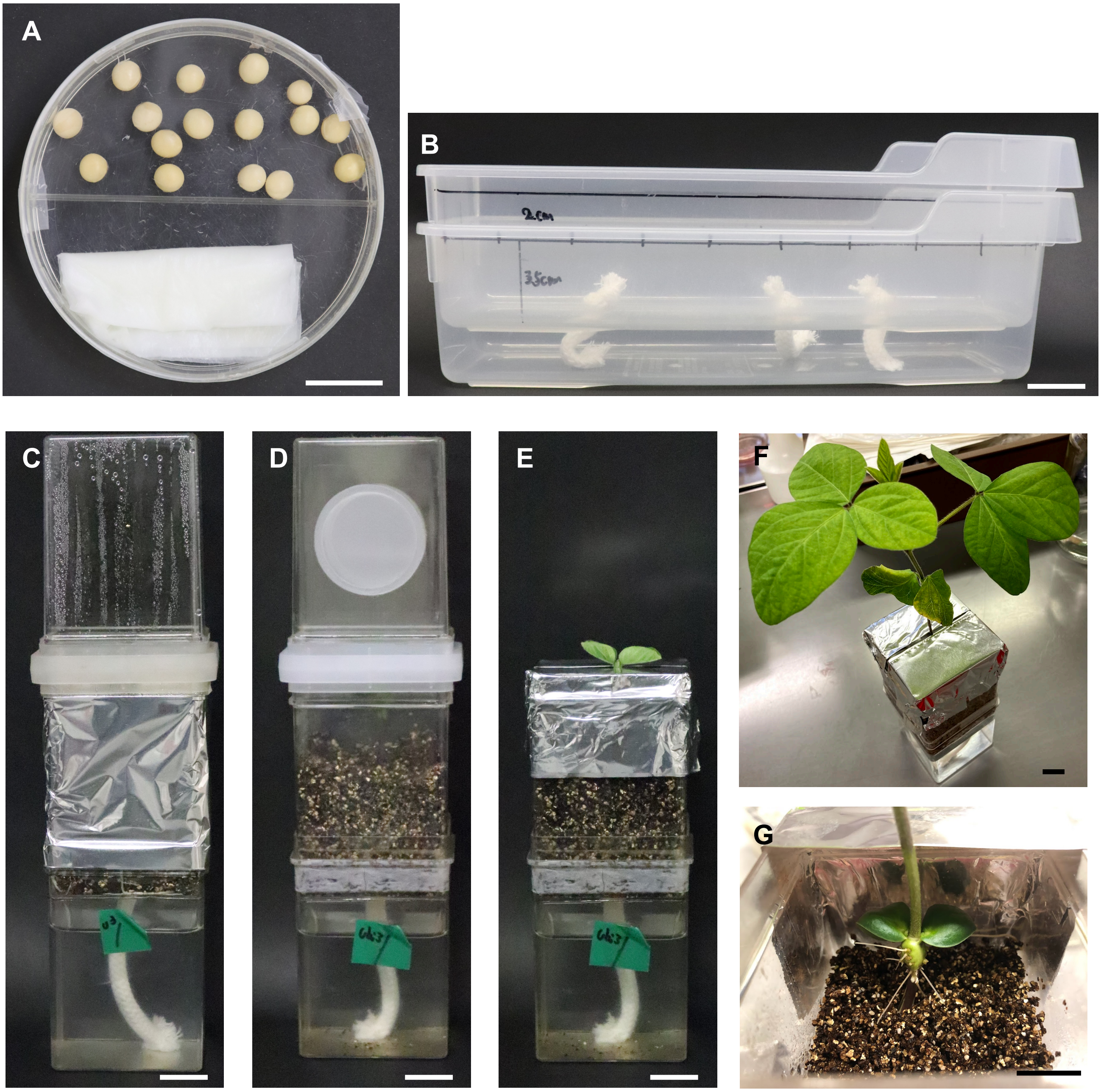

Supplementary Figure S1. Equipments for the hairy root transformation.

A, seed sterilization with vapor of 5% sodium hypochlorite using a separated Petri dish. Seeds were placed in one half of a Petri dish and filter paper soaked in 5% sodium hypochlorite solution was added to the other half. **B**, double-tier container for seed germination. **C**, closed magent box for the entire enclosed plant. **D**, ventilated magent box. **E**, Magenta™ box covering the injection area with aluminum foil. **F**, the plant 17 days after covering the injection area with aluminum foil. **G**, hairy roots to be produced from the injection area covered with aluminum foil 12 days after injection. Scale bars indicate 2 cm.

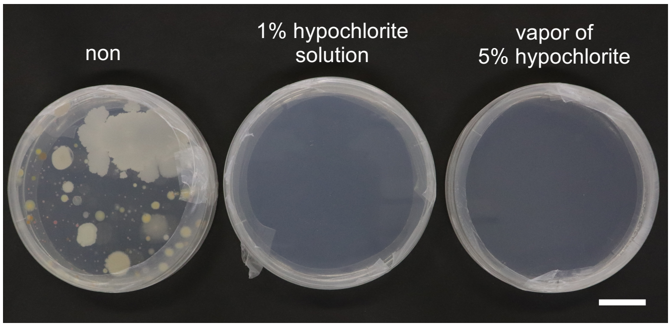

Supplementary Figure S2. Efficiency of seeds sterilization.

Seeds were sterilized with vapor of 5% sodium hypochlorite overnight, sterilized with 1% sodium hypochlorite solution for 1 min, or left non-sterilized. Subsequently, five seeds were rolled on the AG medium with 1.5% agar for 30 s and incubated for 2 days at 28°C. Scale bar indicates 2 cm.

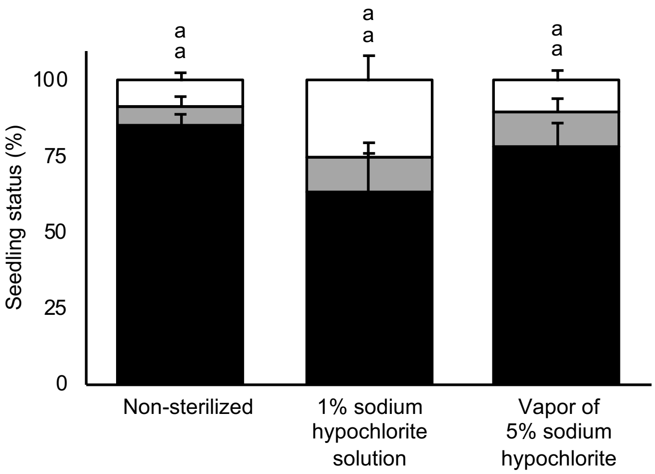

Supplementary Figure S3. Condition of 3-day-seedlings.

Seeds were sterilized with vapor of 5% sodium hypochlorite overnight, sterilized with 1% sodium hypochlorite solution for 1 min, or left non-sterilized. Sterilized seeds were washed with sterile water, spread in a double-tier container, and incubated for 3 days (16 h light/8 h dark) at 28°C. Black bars indicate the ratio of healthy seedlings, gray bars indicate the ratio of slightly damaged seedlings, and white bars indicate the ratio of abnormal germination or non-germination. Means and standard errors represent three independent experiments (n = 20 for each condition). Letters above each bar indicate individual statistically significant differences as determined by Tukey-Kramer's HSD test at $p < 0.05$.

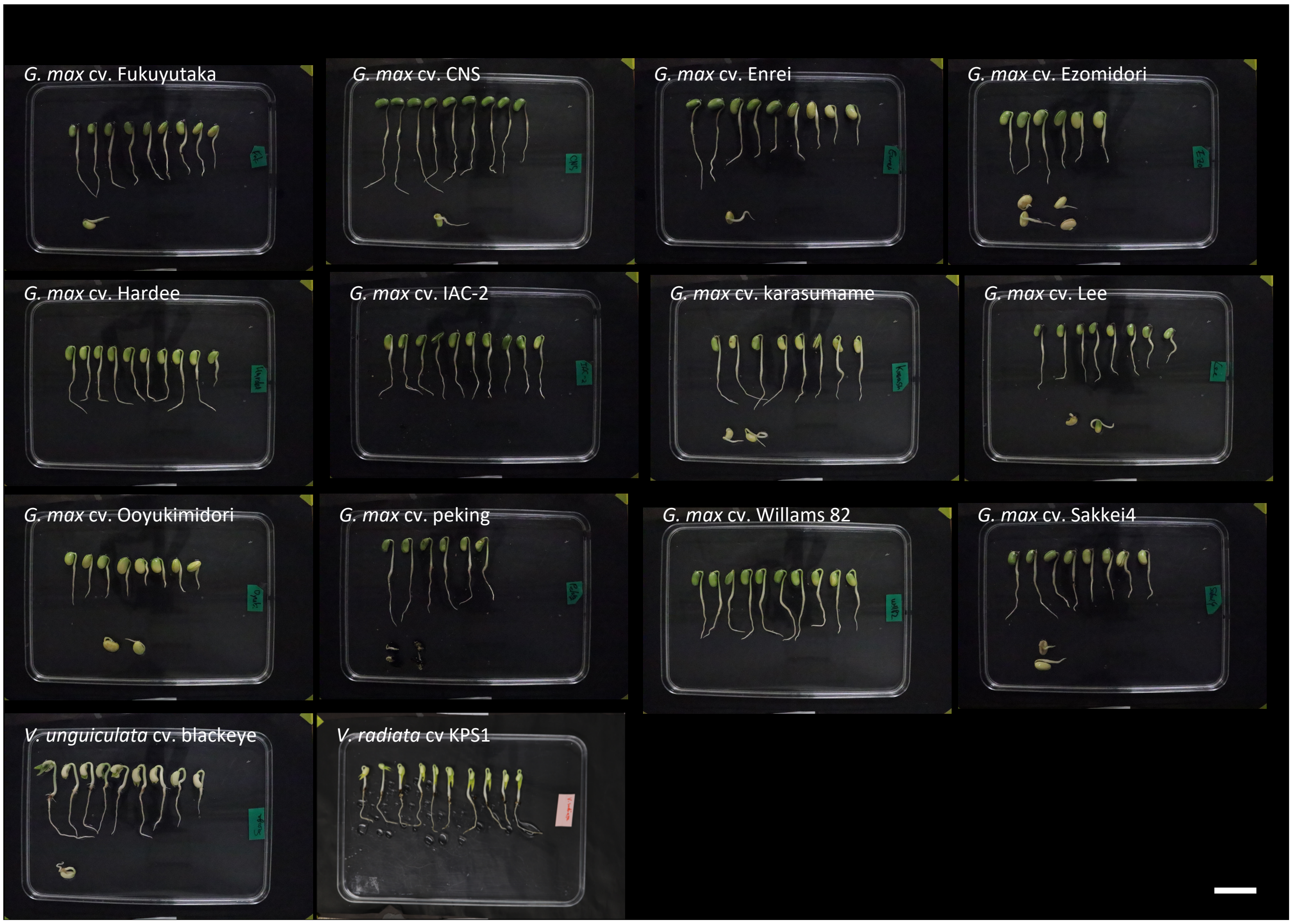

Supplementary Figure S4. Three-days-seedlings of soybean and Vigna cultivars.

Scale bar indicates 5 cm.

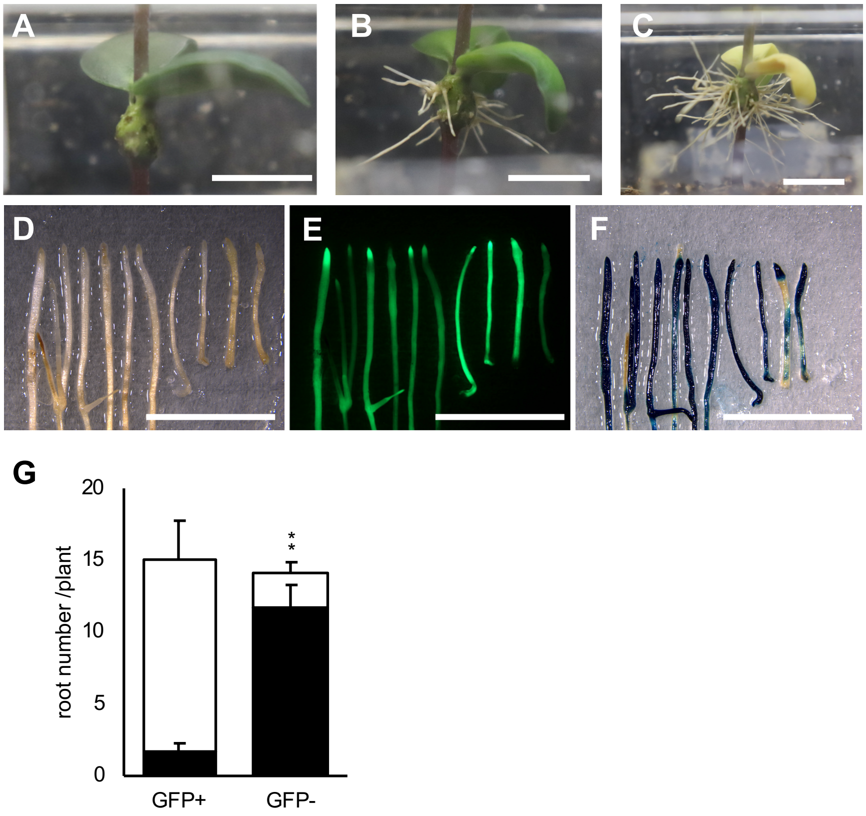

Supplementary Figure S5. Green fluorescent protein (GFP)- and β-glucuronidase (GUS)-based transgenic hairy roots.

A to **C**, hairy root productions after 6 days (**A**), 12 days (**B**), and 18 days (**C**)-post inoculation. Scale bar indicates 1 cm. **D**, transgenic hairy roots, **E**, GFP-expressing hairy roots, **F,** GUS-staining hairy roots. Scale bars indicate 1 cm. **G**, the average of number of GFP-expressing and non-GFP-expressing hairy roots longer than 1cm. white bars indicate GUS-staining hairy roots, and black bars indicate non-GUS-staining hairy roots. Means and standard errors are represented three independent experiments (n = 9). A single asterisk indicates a significant difference between GFP-expression and non-GFP-expression GUS staining results as determined by a Student t-test ($p < 0.05$).

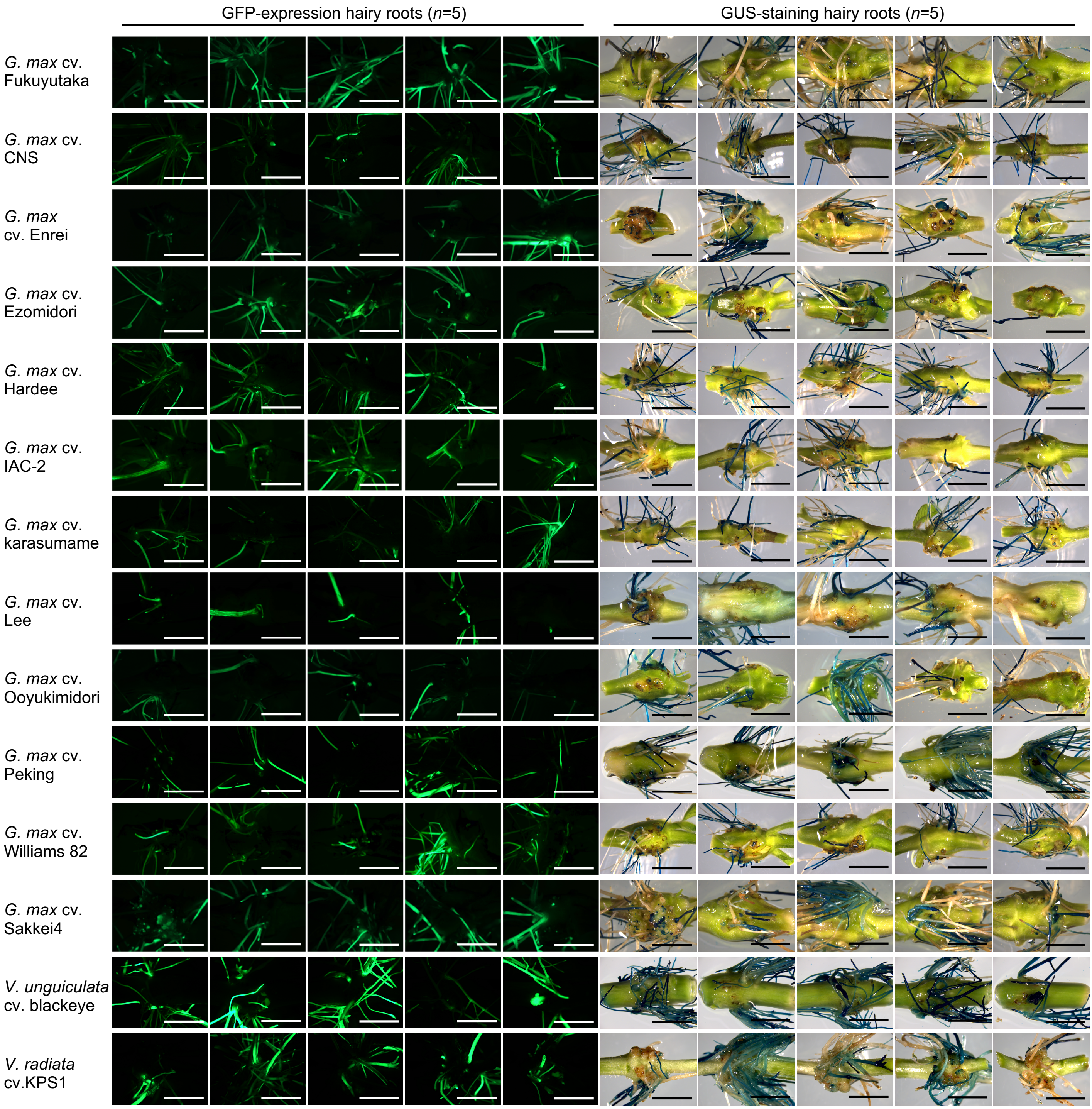

Supplementary Figure S6. Transgenic hairy roots of soybean and Vigna cultivars.

Transgenic hairy roots of 12 soybean, one cowpea, and one mungbean 18 days after *Rhizobium rhizogenes* injection. Each of the five images on the left shows GFP-expression hairy roots, and each of the five images on the right shows GUS staining hairy roots. Scale bars indicate 1 cm.
